# The effectiveness of the use of telehealth programs in the care of individuals with hypertension and, or diabetes mellitus: systematic review and meta-analysis

**DOI:** 10.1186/s13098-022-00846-5

**Published:** 2022-05-28

**Authors:** Daniel Souza Santos, Clara Regina Santos Batistelli, Marina Marilac dos Santos Lara, Emily de Souza Ferreira, Tiago Ricardo Moreira, Rosângela Minardi Mitre Cotta

**Affiliations:** 1 Department of Medicine and Nursing, Federal University of Viçosa (UFV), Viçosa, MG 36570-900 Brazil; 2 Department of Nutrition and Health, Federal University of Viçosa (UFV), Viçosa, MG 36570-900 Brazil

**Keywords:** Diabetes mellitus, Hypertension, Systematic review, Meta-analysis, Telehealth

## Abstract

**Introduction:**

Diabetes *Mellitus* and Hypertension are some of the main Chronic Noncommunicable Diseases, representing a big challenge for global health. In this context, Telehealth programs are presented as a tool with exciting potential to complement and support health care. This paper aimed to analyze the effectiveness of the use of Telehealth programs in the care of individuals with Hypertension and/or Diabetes *Mellitus*.

**Methods:**

A systematic review with meta-analysis was carried out according to the Preferred Reporting Items for Systematic Reviews and Meta-Analyses (PRISMA) protocol methodology. The following databases were used: PubMed, EMBASE, SciELO, ScienceDirect and Cochrane Library. Papers were included if they addressed the use of technologies that allow two-way communication at a distance between health professionals and patients affected by Hypertension and/or Diabetes *Mellitus*, type 1 or type 2. Experimental, cross-sectional, case–control, cohort, and clinical trials were included in the review.

**Results:**

We included 164 papers in the review and 45 in the meta-analysis final synthesis. The systematic review results showed a prevalence of telemonitoring as the main form of Telehealth. The study showed a reduction in expenses with the use of Telehealth, both for the users and for the health systems providers, followed by greater satisfaction. Our meta-analysis showed that Telehealth is an effective tool in the care of diabetic patients, providing a 0.353% reduction in HbA1c compared to traditional care. No studies on Hypertension that met our eligibility criteria for inclusion in the meta-analysis were found.

**Conclusions:**

Telehealth is an effective tool for the care of people with Diabetes *Mellitus* and/or Hypertension.

**Supplementary Information:**

The online version contains supplementary material available at 10.1186/s13098-022-00846-5.

## Introduction

Chronic Noncommunicable Diseases (NCDs) are the leading cause of death in the world, surpassing the number of deaths caused by all communicable diseases. They pose a threat to global health and have been included in the Action Agenda of the Sustainable Development Goals, established in 2015 by the United Nations. In this sense, the goal of reducing premature mortality from NCDs by one-third was set by 2030, through prevention, treatment, and well-being promotion [1–3].

Therefore, Diabetes *Mellitus* (DM), one of the main NCDs, represents one of the greatest challenges, as a growth of half a billion cases of the disease is expected by the end of the decade, associated with an increase in premature deaths caused by this condition, while the trend in most other NCDs is of decline. Currently, approximately 6% of the world's population lives with DM, either type 1 or 2, at risk of serious and irreversible complications without adequate diagnosis and management [2].

This also occurs in Hypertension (AH), which has a high prevalence in the population and can lead to severe cardiovascular and coronary diseases if not treated correctly. In this case, diagnosis and treatment are usually simple, but the often-asymptomatic character of the disease makes it difficult to add to it. Thus, the worldwide control of AH is still weakened, and for every 5 individuals with the disease, less than 1 is managed [4, 5].

Therefore, with the emergence of the first cases of individuals affected by COVID-19, health systems suffered even more from the interruption of non-urgent services to assist in responding to the pandemic and containing the spread of the novel coronavirus. Added to this is the vulnerability of individuals with NCDs in the face of this new disease, which reflected in high rates of people with DM and/or AH hospitalized and with severe manifestations of COVID-19 [1, 2]. In this regard, Telehealth programs, which consists of the use of Information and Communication Technologies by health professionals, to assist the population through disease and disease prevention, diagnosis, treatment, and health education, is presented as a tool with exciting potential to complement and support care. Thus, the development of well-planned digital solutions is essential to improve access and results for population groups of individuals with NCDs [2, 6–9].

Thus, this study aimed to analyze the effectiveness of the use of Telehealth programs in the care of individuals with AH and, or DM.

## Methods

### Study design

This article is a systematic review (SR) and meta-analysis.

#### Protocol and registration

This SR was planned and executed according to the Preferred Reporting Items for Systematic Reviews and Meta-Analyses (PRISMA) protocol methodology [10] and registered with the International Prospective Register of Systematic Reviews (PROSPERO), under protocol number CRD42020215527.

#### Search and research sources

Five databases were selected in which the research was conducted to identify and select potentially relevant studies for the development of SR. The databases selected for the initial search were the following ones: Medical Literature Analysis and Retrieval System Online (MEDLINE/PubMed), Excerpta Medica dataBASE (EMBASE), Scientific Electronic Library Online (SciELO), ScienceDirect and Cochrane Library. The date stipulated for the search was January 1st, 2000, to May 5th, 2021, this being the date of the last search. The exclusion of articles dating before to January 2000 is due the fact that communication technologies used prior to this date no longer represent the current reality of telemedicine [11].

The descriptors and Booleans used to perform the search were: "telemedicine" OR "Telehealth" OR "mHealth" OR "mobile health" AND "hypertension" OR "diabetes mellitus". The studies identified in the databases mentioned were conducted through the StArt™ (State of the Art through Systematic Review) program, to facilitate the selection of the articles included in the SR.

#### Study selection criteria

For the SR, we have included original studies, conducted from January 2000, without restriction regarding the published language or place of study, where the study addressed the use of technologies that allow two-way communication from a distance between health professionals and patients affected by AH and, or Type 1 or Type 2 DM. Experimental, cross-sectional, case–control, cohort, and clinical trials were included in the review.

Non-original studies and studies where it was not possible to extract data relevant to the analysis were excluded, such as letters, editorials, congress annals, comments, reports, study protocols, pilot studies, abstracts, and reviews. In addition, we excluded articles that did not address a population diagnosed with DM or AH, studies on teleconsulting among health professionals, and studies where the technology presented did not allow the double path of communication entirely from a distance.

For the inclusion of the studies in the meta-analysis, we selected studies that compared the use of Telehealth programs with usual care and that there were pre- and post-intervention data so that we could estimate the effectiveness of Telehealth programs in the target population. In addition, it was necessary that studies on the population with DM necessarily obtained data regarding glycated hemoglobin (HbA1c) and standard deviation, before and after the intervention, for the intervention and control groups. For the population with AH, it was necessary to obtain data from systolic pressure and standard deviation, before and after the intervention, for the intervention and control groups.

#### Screening process

The authors screened the titles and abstracts independently, and later the discrepancies were discussed and summarized. In this first phase, duplicate studies extracted from different databases were excluded. Subsequently, we identified potentially eligible articles, according to the inclusion and exclusion criteria, by reading the titles of the articles. We applied the same criteria for the second phase, which was the selection based on the reading of the abstracts. The studies screened for full reading were independently analyzed by all authors and the divergences were discussed for the final selection of the articles elected to integrate the systematic review.

#### Data extraction process

The authors used a standardized file to extract useful data for the SR. The data extracted were title, name of the first author, year of publication, year of research, country of study, type of study/design/design, number of study participants, data collection instrument, level of health care, disease(s) studied, sample characteristics, characteristic of the Telehealth programs intervention and main study results. The data extracted from all the elected studies were conducted using the Microsoft^TM^ Excel program.

#### Evaluation of the quality of studies

The methodological quality of the articles studied was measured using critical evaluation tools of the Joanna Briggs Institute [12], according to each type of study—cohort, randomized, cross-sectional, and quasi-experimental clinical trial. The results were calculated in percentage, scoring for 1 point for "YES", 0.5 point for "NOT CLEAR" and 0 for "NO". The studies that scored above 75% were considered of excellent quality [13]. Quality has not been established as an exclusion criterion.

### Statistical analysis

Meta-analysis was performed using random fixed-effect models (when necessary) in the Stata® program (version 11.0). Heterogeneity was evaluated by the chi-square test (χ^2^) with a significance of 90% (*p* < 0.10), and its magnitude was determined by the I-square (I^2^) [14]. Thus, heterogeneity was classified as low, moderate, or high when the I-square values were above 25, 50, and 75%, respectively.

In addition, the dispersion of individual results in the forest plot was also used to visually evaluate the presence of statistical heterogeneity. The analyses were performed with the “Metan” and “Metareg” commands, and meta-regressions were performed with the objective of identifying the causes of heterogeneity, using the Knapp and Hartung test [15]. Initially, a univariate analysis was performed. All variables associated with the risk of treatment abandonment in this analysis (*P* ≤ 0.20) were included in the final multivariate model. For these analyses, a significance level of 5% was established. The existence of the small-study effect was also evaluated by visual inspection of the funnel graph and Egger test [16].

The results were synthesized by using meta-analysis from the HbA1c means in the post-test sample of the intervention and control group and respective standard deviation. The choice of post-test means occurred since the groups were well balanced (i.e., similar means) at the beginning of the studies (pre-test).

A random-effect model was used to calculate the absolute difference between means for each result found. The statistical significance of the size of the overall effect of the use of Telehealth programs was determined by the confidence interval (CI) of 95% and significance level of 5%. All analyses were performed in Stata^®^ software version 11.

## Results

### Searches and selection of studies

The search in the selected databases generated a total of 14,686 studies, and of these, 12,669 remained after the exclusion of duplicate studies between different databases. After screening based on the reading of the title, 1,507 studies remained eligible for reading the abstracts. Of the remaining studies, 551 remained eligible after screening by abstracts. Among the articles read in full, 164 met the eligibility criteria and entered the SR. Of these, 45 were chosen for meta-analysis.

**Fig. 1:**
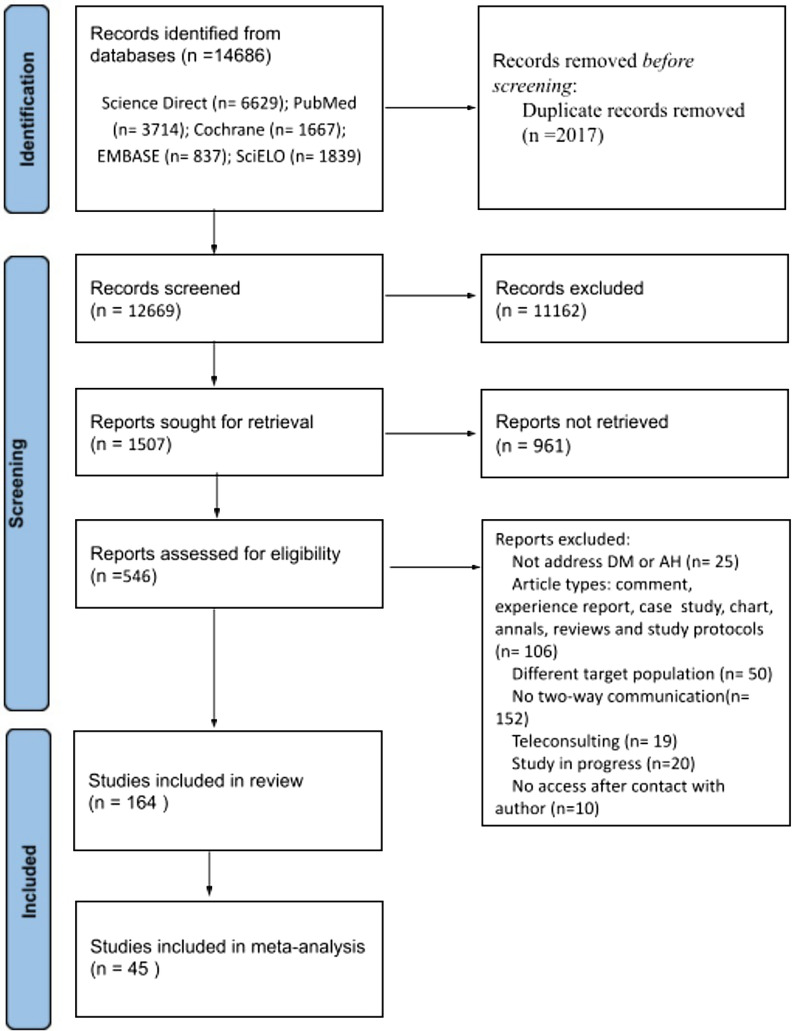
Flowchart of bibliographic research and selection of studies for systematic review

Four studies were excluded because we could not access the full text, even after requesting it to the corresponding authors. The flowchart below illustrates the process of selecting the studies (Fig. 1).

### Characteristics of the studies

Among these studies, 127 addressed the use of Telehealth programs in the care of patients with type 1, 2, or both DM, 33 studies addressed AH and 4 addressed both health conditions.

Regarding the type of Telehealth service studied, 23 addressed teleconsultation, 14 were related to tele-education in health, and 73 studied telemonitoring. Also, 1 study addressed a teleconsultation service with tele-education, 13 approached teleconsultations with telemonitoring, 28 involved tele-education with telemonitoring, and 4 studies addressed teleconsultation, tele-education, and telemonitoring.

Regarding the place of study, 72 were from North America, 2 from South America, 6 from Africa, 36 from Europe, 33 from Asia, and 8 from Oceania. Only 7 studies failed to mention the location where they had been carried out in their bodies.

An additional file shows all studies selected for the SR with their characteristics. [see Additional file 1].

### Meta-analysis

**Fig. 2:**
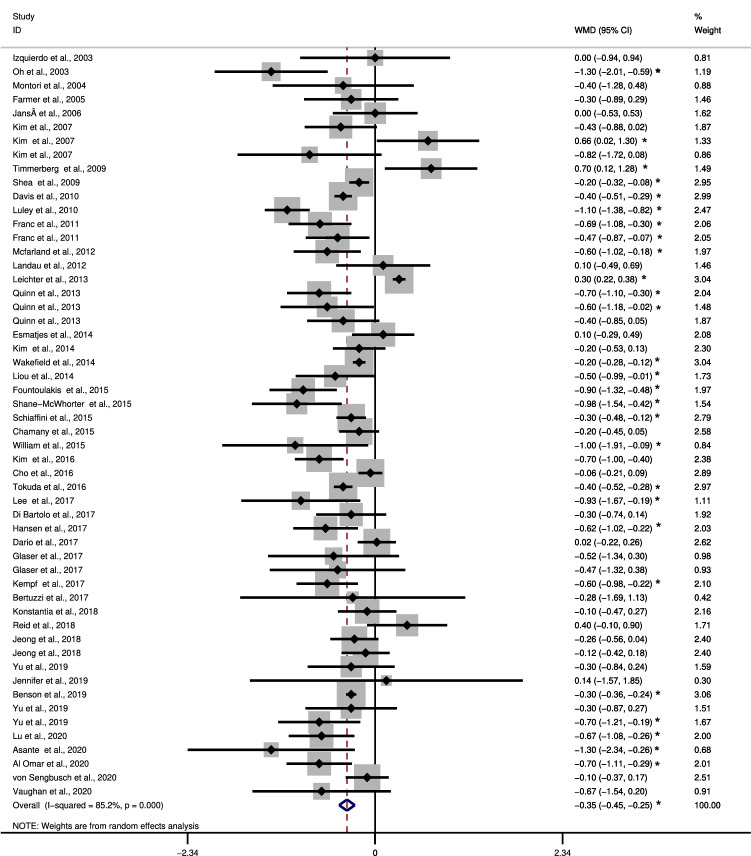
Meta-analysis. *statistically significant results, *WMD *weighted mean differences, Weights are from random effects analysis, Heterogeneity chi-squared = 358.87 (d.f. = 53) p = 0.000, I-squared (variation in WMD attributable to heterogeneity) =  85.2%

According to Fig. 2, the 45 eligible articles appear in order of publication and have weighted average value of reduction or increase of glycated hemoglobin, a confidence interval, and a weight that varies according to the sample size of the article. This figure shows that most studies indicate that the intervention group was significantly better in relation to the control group regarding to the outcome, which would be the drop in glycated hemoglobin.

Only three studies showed that the control group obtained better results than the intervention group and 27 studies failed to present results regarding the groups. As the main result, we found a difference of 0.353 between the intervention and control groups, indicating that the Telehealth group had a significant reduction in HbA1c (%) value compared with the usual care group, an improvement of -0,353%. No meta-analysis eligible study addressed hypertension.

In addition, there was high heterogeneity among the studies (85.2%) (*P* < 0.001) which indicates a great variation in the results. We tried to investigate the reasons for this high variation by performing subgroup analysis and meta-regression, but the reasons for heterogeneity were not found among the variables investigated.

**Fig. 3:**
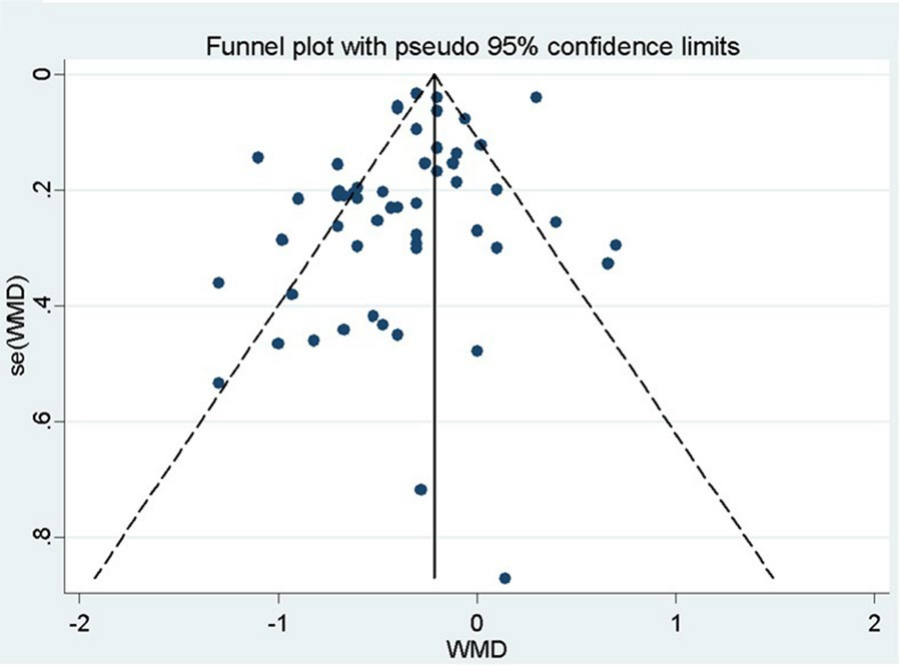
Graph of Funnel. *WMD *weighted mean differences, Funnel plot with 95% confidence limits

Figure 3 shows the Meta Funnel, a test performed to investigate the risk of publication bias. In our analysis, although most studies are within the delimitations of the funnel, we still obtained studies outside these delimitations, which indicates a risk of bias. The Egger test was also performed, which indicated significant results (*P* = 0.024) reinforcing the results obtained by the Meta Funnel test.

### Quality of studies

Fifty-three studies scored less than 75% in the quality evaluation criteria being considered of low quality. The other 111 studies were considered of high quality according to the criteria adopted in this study. Quality results can be found in the supplementary file [see Additional file 1].

## Discussion

Our study showed that Telehealth programs are an effective tool in the approach of individuals diagnosed with DM, reducing the HbA1c by 0,353% more than face-to-face care. Approximately 77% of the SR were studies related to the care of people with DM. Telemonitoring, which is the use of information technology to monitor patients from a distance, was the most recurrent Telehealth axis among the included studies, corresponding to 44.5% of the studies, being succeeded by tele-education, associated or not with other Telehealth axes. There was a significant predominance of studies conducted in North America in relation to other continents. The European and Asian continents also stood out in relation to the number of studies from South America, Africa, and Oceania.

According to a recent report, published in 2018 by the American Diabetes Association (ADA), the application of telemedicine in diabetes is related to a considerable improvement in glycemic management [17]. Our meta-analysis showed that Telehealth programs are an effective tool in the care of diabetic patients, providing a greater reduction of glycated hemoglobin compared to the usual care. Such results are in line with other studies that also evaluated these characteristics [18–22]. No studies on AH were found that met our eligibility criteria for inclusion in the meta-analysis.

In our review, the use of Telehealth programs reduced HbA1c by 0.353% in relation to traditional (face-to-face) care. Another SR and meta-analysis found related results, with an improvement between − 0.37% to − 0.55% in HbA1c in relation to the usual care [23]. In addition, other studies have found results like ours, pointing to Telehealth as an effective strategy for the care of patients with DM [23, 24].

Although it was not possible to perform the meta-analysis of studies on AH, most studies included in the SR found satisfactory results regarding the use of Telehealth programs in the care of people diagnosed with AH. In addition, guidelines from the European Society of Cardiology (ESC) and the European Society of Hypertension (SEH) point out that for the management of AH telemonitoring can result in better results for patients affected with this pathology [25]. Other SR [26–28] studied the isolated effectiveness of different forms of Telehealth programs and found positive results in relation to the usual care.

In our study, 72 of the 164 of the studies found were conducted in North America, 36 in Europe and 33 in Asia. Another review [29] found a similar result, with the North American continent presenting a prevalence of studies on Telehealth programs. In general, the discrepancy in the number of Telehealth services offered in separate locations in the world are influenced by political, technological, legal, financial, business strategy and human resources factors [30]. Thus, localities where internal policy promotes the technological and health system development, there is an environment of technological innovation, laws and standards that allow and regulate telemedicine, public or private investment in the area and qualified professionals in the use of the Telehealth tools, greater number of health services are developed with the use of Information and Communication Technologies, such as North America and Europe [30, 31].

Our SR also highlighted a tendency to reduce expenses with health services when Telehealth was used, both for the users and for the health care providers [32–36]. Deng et al. found a reduction of approximately 37.8% in annual costs with the intervention of Telehealth program [37]. Although the short-term cost-effectiveness ratio may be questionable, due to the high initial cost for domestic telemonitoring, it is suggested that the investment would be recovered in the long term [38, 39]. Added to this is the high degree of patient satisfaction with the use of Information and Communication Technologies for the care of DM and AH found in several studies, emphasizing its efficiency [40–43].

A high level of heterogeneity was found in the analysis of the results and could not be explained by subgroup analysis and meta-regression. Two explanations for heterogeneity are variability between participants and methodological variability. The variability among the participants may be due to the differences that each country has from the point of view of socioeconomic, cultural, and disease profile. Regarding methodological variability, we observed some studies with small samples and different forms of application of the Telehealth programs axes, as well as different inclusion criteria that may have influenced these differences between them.

The limitations found in the study are as follows: high heterogeneity and the fact that we did not find articles on AH that contemplated the inclusion criteria and presented systolic blood pressure means in the sample post-test of the intervention and control groups and respective standard deviation, for inclusion in the meta-analysis.

Our paper has compiled new and relevant information. It showed that Telehealth presents lower cost, while it is a useful tool in the care of patients with DM and/or AH with great acceptance. Furthermore, we proved that Telehealth is more effective than usual care in the reduction of HbA1c. Finally, this paper highlighted the need for regulation of Telehealth and health policies aimed at fostering technological innovation and development, especially in locations where Telehealth is not widespread as a form of care for patients with chronic noncommunicable diseases.

## Conclusion

The findings of this systematic review and meta-analysis confirmed the Telehealth programs as an effective tool for the care of people with DM and, or AH. This study brought new and relevant information regarding the care of patients with DM or AH with the use of Telehealth. It is recommended to conduct further studies that evaluate the health of people with AH and that individually evaluate diverse sources of Telehealth programs in the care of patients with DM and/or AH, emphasizing the need for further studies in the African and South American continents.

## Supplementary Information

**Additional file 1: **“Selected studies for the SR”. It shows all the studies selected for the SR, with their respective titles, authors, year of publication, country of study, type of Telehealth, number of participants in the study, disease addressed and the level of quality of the study. File format: .xls.

## Data Availability

Not applicable.

## Abbreviations

AH: Hypertension
DM: Diabetes *mellitus*
EMBASE: Excerpta Medica dataBASE
MEDLINE: Medical literature analysis and retrieval system online
NCD: Noncommunicable chronic diseases
PRISMA: Preferred Reporting Items for Systematic Reviews and Meta-Analyses
PROSPERO: International Prospective Register of Systematic Reviews
SciELO: Scientific Electronic Library Online
SR: Systematic review
StArt: State of the art through systematic review
